# Functional Expression of Spider Neurotoxic Peptide Huwentoxin-I in *E. coli*

**DOI:** 10.1371/journal.pone.0021608

**Published:** 2011-06-23

**Authors:** Er Meng, Tian-Fu Cai, Wen-Ying Li, Hui Zhang, Yan-Bo Liu, Kuan Peng, Songping Liang, Dong-Yi Zhang

**Affiliations:** 1 Key Laboratory of Protein Chemistry and Developmental Biology of the Ministry of Education, College of Life Sciences, Hunan Normal University, Changsha, Hunan, China; 2 Research Center of Biological Information, College of Science, National University of Defense Technology, Changsha, Hunan, China; 3 Core Facilities of Biotechnology, Central South University of Forestry and Technology, Changsha, Hunan, China; University of Crete, Greece

## Abstract

The coding sequence of huwentoxin-I, a neurotoxic peptide isolated from the venom of the Chinese spider *Ornithoctonus huwena*, was amplified by PCR using the cDNA library constructed from the spider venom glands. The cloned fragment was inserted into the expression vector pET-40b and transformed into the *E. coli* strain BL21 (DE3). The expression of a soluble fusion protein, disulfide interchange protein (DsbC)-huwentoxin-I, was auto-induced in the periplasm of *E. coli* in the absence of IPTG. After partial purification using a Ni-NTA column, the expressed fusion protein was digested using enterokinase to release heteroexpressed huwentoxin-I and was further purified using RP-HPLC. The resulting peptide was subjected to gel electrophoresis and mass spectrometry analysis. The molecular weight of the heteroexpressed huwentoxin-I was 3750.69, which is identical to that of the natural form of the peptide isolated from spider venom. The physiological properties of the heteroexpressed huwentoxin-I were further analyzed using a whole-cell patch clamp assay. The heteroexpressed huwentoxin-I was able to block currents generated by human Na_v1.7_ at an IC_50_ of 640 nmole/L, similar to that of the natural huwentoxin-I, which is 630 nmole/L.

## Introduction

**Figure 1:**
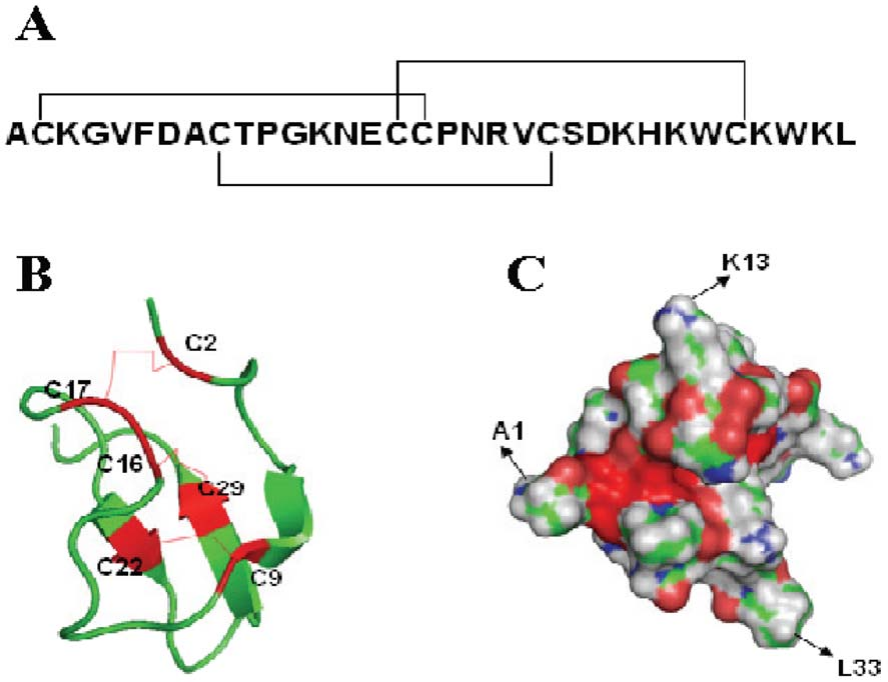
Characterization of HWTX-I. A) Amino acid sequence and disulfide bonds (black lines) of HWTX-I. B) 3D structure of HWTX-I (PDB entry 1qk6). All of the cysteines and disulfide bridges are shown in red. C) Model of the surface calculation of HWTX-I (version 0.99 beta37 by PyMOL).

Huwentoxin-I (HWTX-I) is a neurotoxic peptide isolated from the venom of the Chinese bird spider *Ornithoctonus huwena*, which is distributed in the hilly areas of the provinces of Yunnan and Guangxi in southern China. The primary structure of HWTX-I was previously determined. It consists of 33 amino acid residues and three pairs of disulfide bonds [1], [2]. Spatial structure analysis demonstrated that HWTX-I adopts a compact structure consisting of a small triple-stranded antiparallel β-sheet stabilized by three disulfide bonds (Fig. 1) [3]. HWTX-I possesses multiple biological activities. It reversibly blocks neuromuscular transmission in an isolated mouse phrenic nerve-diaphragm preparation. It was demonstrated that HWTX-I is a toxin that blocks N-type voltage-gated calcium channels (VGCCs) and TTX-_S_ voltage-gated sodium channels (VGSCs) in adult rat dorsal root ganglion (DRG) neurons [4], [5], [6]. It has also been reported that in a rat formalin test model, the intrathecal administration of HWTX-I is effective in antinociception [7]. Therefore, HWTX-I has been considered a model molecule for anti-pain drug development and is currently in phase I clinical trials.

Although HWTX-I is the most abundant component in venom (about 15% of the total protein, w/w), isolating HWTX-I from crude venom using biochemical tools cannot meet the increasing demands of research due to the limited venom supply and purification costs. The ultimate solution to this problem would be the efficient expression of recombinant HWTX-I (rHWTX-I) in prokaryotic organisms, such as *E. coli*, or eukaryotic systems, such as yeast or cultured cells. In previous attempts, a synthesized nucleotide fragment was used to express rHWTX-I fused with either glutathione S-transferase (GST) or ketosteroid isomerase (KIS) in *E. coli* cells. The fusion proteins were expressed in the cytoplasm of *E. coli*. rHWTX-I released from the fusion protein demonstrated extremely low bioactivity before a reduction/renaturation treatment. Although the reduction/renaturation treatment was able to fully restore the bioactivities of rHWTX-I, the average yield went down to less than 50 µg/L [8], [9]. Expressing rHWTX-I in the yeast system *Pichia pastoris* was also attempted. Four additional amino acid residues were attached to the N-terminal of the expressed rHWTX-I, and the bioactivity of the expressed peptide was only 70% in comparison with that of the natural toxin [10]. A baculovirus system was also used for the expression of rHWTX-I, but neither the yield nor the cost was satisfactory, despite the fact that the expressed peptide demonstrated natural bioactivities [11]. In summary, no efficient system has been developed thus far to express rHWTX-I in a way that maintains natural activities with a satisfactory yield.

In this report, we inserted a cDNA copy of HWTX-I into the pET40b expression vector. rHWTX-I was expressed in fusion with DsbC in the periplasm of *E. coli* BL21 (DE3) cells. rHWTX-I was conveniently purified in a Ni-NTA column and then subjected to enterokinase digestion. After RP-HPLC purification, the resulting rHWTX-I demonstrated identical properties to the natural toxin both biochemically and physiologically.

## Materials and Methods

### Materials

The *E. coli* strain Top10F' used for plasmid cloning was purchased from Invitrogen (Carlsbad, CA, USA). The expression vector pET-40b and the *E. coli* host strain BL21 (DE3) were purchased from Novagen (Madison, WI, USA). The human embryonic kidney 293 (HEK293) cell line was purchased from the Cell Resource Center (Shanghai Institutes for Biological Sciences, China Academy of Sciences). Enterokinase was purchased from Majorbio (Shanghai, China). All restriction enzymes and other enzymes used in molecular cloning experiments were purchased from Fermentas (Burlington, ON, Canada) if not otherwise indicated. All chemicals and reagents were purchased from Sigma (St. Louis, MO, USA). The synthesis of primers and the DNA sequencing of the constructed plasmids were performed by Sangon (Shanghai, China). The venom gland cDNA library of the spider *Ornithoctonus huwena* was previously constructed and kept in Prof. S. Liang's laboratory.

### Construction of pET40b-rHWTX-I plasmid

Based on the cDNA sequence of HWTX-I (GenBank Accession No. AY 263711) [12], two primers were designed to amplify the coding sequence of HWTX-I. The P-huwen-I-upper primer (5′-CCGGAATTCCGCGTGCAAAGGGGTTTTTGATG-3′) contains an *EcoR I* restriction site (underlined), whereas the P-huwen-I-lower primer (5′-CCGCTCGAGTTATAATTTCCATTTACACCACTTGTG-3′) contains a *Xho I* restriction site (underlined). Using the venom gland cDNA library as the template, the HWTX-I gene was obtained by PCR and inserted between the *EcoR I* and *Xho I* sites present in pET40b. The resulting plasmid was named pET40b-FrHWTX-I.

**Figure 2:**
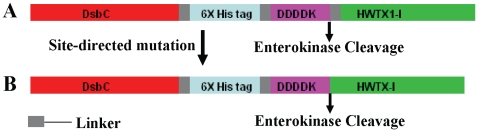
Construction of the pET40b-rHWTX-I expression vector. A) pET40b-FrHWTX-I. B) pET40b-rHWTX-I.

In the plasmid pET40b-FrHWTX-I, 45 additional bases were present between the enterokinase cleavage site and the HWTX-I gene, which would add 15 extra amino acid residues to the N-terminal of HWTX-I if expressed. Following a site-directed deletion mutation procedure described before [13], the additional bases were eliminated and the resulting plasmid was named pET40b-rHWTX-I (Fig. 2). The DNA sequences of all constructed plasmids were confirmed by DNA sequencing carried out by Sangon (Shanghai, China).

### Expression and purification of rHWTX-I

The expression vector pET40b-rHWTX-I was transformed into the *E. coli* strain BL21 (DE3). Single colonies from the transformants were inoculated in a 5 ml ZYM-505 medium containing 100 µg/ml kanamycin, which was shaken at 200 rpm at 37°C. After 6–8 hours of incubation, the OD_600nm_ of the culture reached about 0.8 (turbid yet not saturated). Then, 400 µl of the culture was transferred into an 800 ml fresh ZYM-5052 medium (containing 100 µg/ml kanamycin). According to an IPTG-free auto-induction method of protein expression in the T7 system reported before [14], the mixture was incubated overnight at 25°C at a shaking rate of 200 rpm. The cells were harvested by centrifugation at 4200 g for 15 min and the supernatant was decanted. After suspending the cells in sucrose-EDTA solution (30 mM Tris-HCl, pH 8.0, 20% sucrose, 1 mM EDTA), the periplasmic fractions were prepared by osmotic shock as previously described [15].

The periplasmic fractions were applied to a gravity Ni-NTA column, and the eluents were subjected to dialysis to adjust to a condition suitable for enterokinase digestion. Enterokinase digestion was then performed for 16 hours at 23°C to remove the DsbC part of the fusion protein. The digests were centrifuged at 17000 g for 15 min to remove insoluble particles, and the solution was subjected to another round of Ni-NTA column purification. The digested rHWTX-I flowed through the column while DsbC and enterokinase (which also contained a His-tag) were captured by the column. The collected flow-through was subjected to RP-HPLC purification using a C18 column (4.6 mm×250 mm), and a linear acetonitrile gradient of 25–35% was used for the purification.

### Glycine-SDS-PAGE gel electrophoresis

Glycine-SDS-PAGE analysis of the cell lysates, partially purified proteins, and purified proteins was performed according to the Laemmli method using 12% acrylamide gels [16].

### MALDI-TOF/TOF mass spectrometry

A MALDI TOF/TOF MS spectrometer (Ultraflex™, Bruker Daltonics) was used to determine the molecular mass and purity of the purified rHWTX-I, according to a method reported previously [17].

### Whole-cell patch-clamp experiments

Whole-cell patch-clamp experiments were carried out according to a protocol described by Xiao et al. [18]. Briefly, cultured human embryonic kidney 293 (HEK293) cells were transiently transfected with a plasmid harboring the cDNA fragment encoding human Na_v1.7_ (a subtype of human VGSC α subunits). At 36–72 hours after the transfection, cells emitting green fluorescence were selected for whole-cell patch-clamp recordings. Purified rHWTX-I and natural HWTX-I were dissolved in distilled water to a concentration of 1 mmole/L and stored at −20°C. Before use, the stock solution was diluted to the concentrations of interest with fresh bathing solution. Whole-cell patch-clamp assay recordings were performed at room temperature using an EPC-9 amplifier (HEKA, Lambrecht, Germany). Sodium currents were elicited at −10 mV from a holding potential of −80 mM. The recording and analysis of data were carried out using the Pulse+Pulsefit 8.0 (HEKA, Lambrecht, Germany) and the Sigmaplot 9.0 (Systat Software Inc.) programs, respectively.

## Results

### Gene expression and purification of rHWTX-I

**Figure 3:**
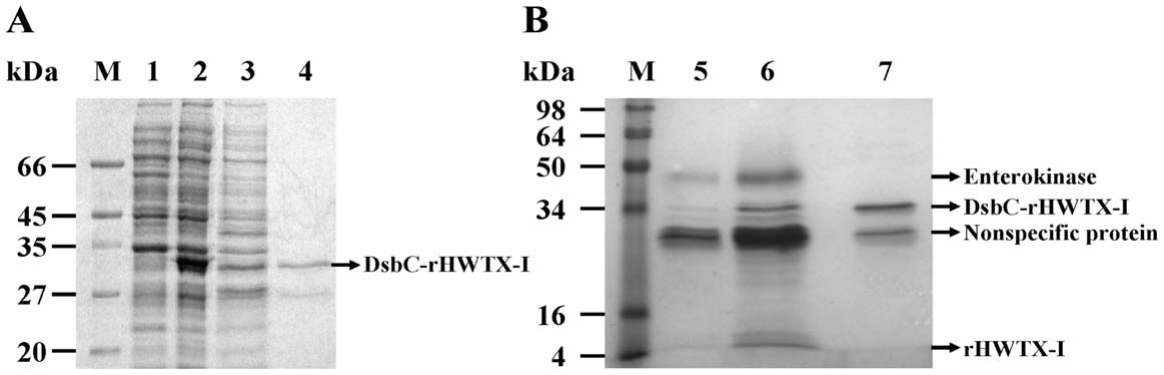
SDS-PAGE analysis. A) Lane M, protein molecular weight markers; lane 1, uninduced cell sample; line 2, induced cell sample; line3, total soluble proteins extracted from the periplasm of induced cells; line 4, purified fusion protein (arrow indicates DsbC-rHWTX-I). B) Lane M, protein molecular weight markers; lane 5, 20 µl DsbC-rHWTX-I after enterokinase digestion; lane 6, 100 µl DsbC-rHWTX-I after enterokinase digestion; line 7, negative control (10 µl DsbC-rHWTX-I, enterokinase free).

The gel electrophoresis analysis of expressed and purified rHWTX-I is shown in Figure 3. As shown in Figure 3A, compared with the uninduced cell sample (lane 1), a new band appeared in the auto-induced cell sample (lane 2); the estimated molecular weight of this new band was about 34 kDa, which was consistent with the theoretical molecular weight of the DsbC-HWTX-I fusion protein. After osmotic shock, the soluble periplasm extracts (lane 3) were subjected to Ni-NTA column purification, which led to the partial purification of the 34 kDa product (lane 4). The semiquantitative SDS PAGE analysis demonstrated that the yield of the fusion protein was approximately 10 mg/L.

After a dialysis procedure (15 K MWCO), the desalted fusion proteins were subjected to enterokinase digestion. The digests were assayed using SDS-PAGE followed by Coomassie Blue staining. As shown in panel B of Figure 3, enterokinase digestion of the partially purified fusion protein (lane 7) released two major products (lane 6). The molecular weights of the resulting products were 30 kDa and 4 kDa, representing the DsbC protein and free HWTX-I, respectively.

**Figure 4:**
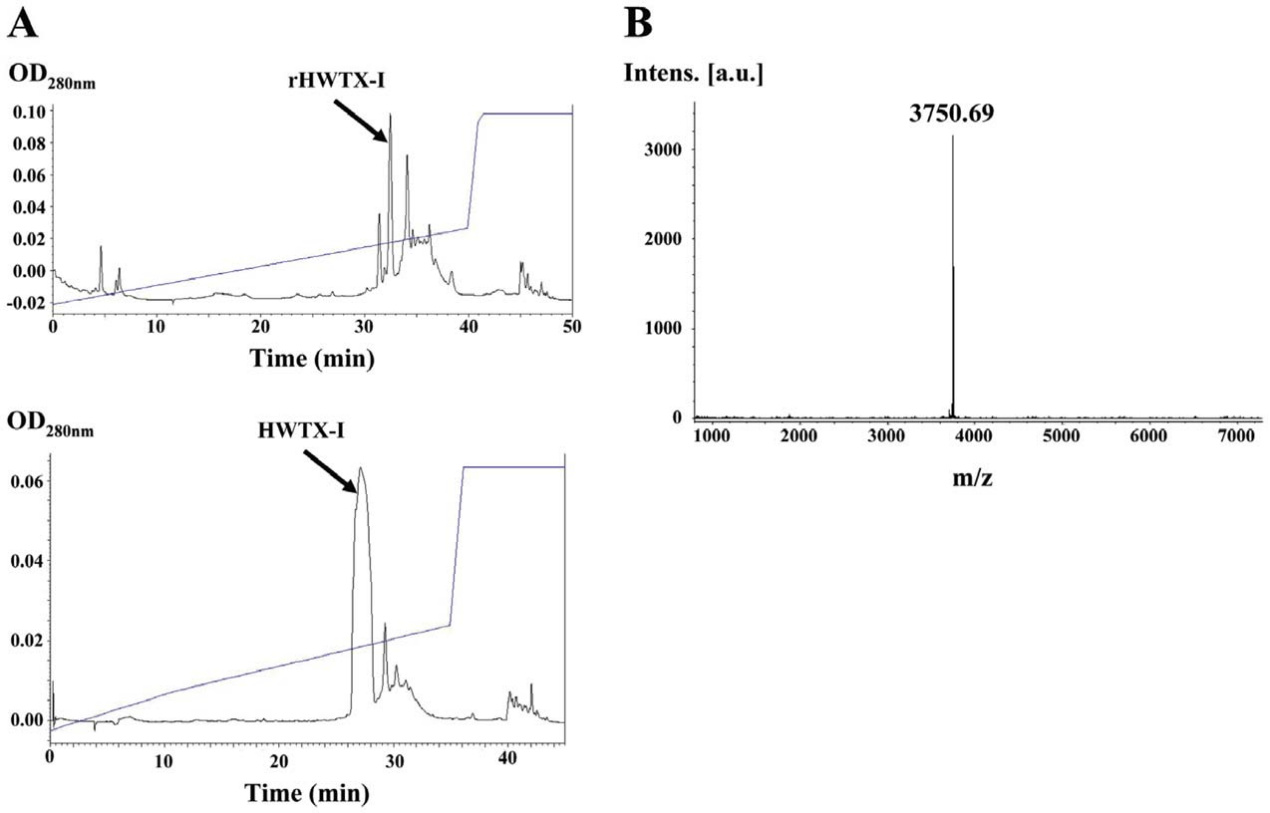
RP-HPLC chromatography and mass spectra of rHWTX-I. A) RP-HPLC chromatography of rHWTX-I (upper) and native HWTX-I (lower) in the same C18 column. Elution was monitored at 280 nm. rHWTX-I and native HWTX-I were eluted at an acetonitrile concentration of 30.4% and 30.6%, respectively. B) Mass spectra of purified rHWTX-I. The calculated theoretical molecular weight of rHWTX-I was 3756.45 Da and the measured molecular weight was 3750.69 Da.

The RP-HPLC purification profile of rHWTX-I released after enterokinase digestion is shown in Figure 4A. Heteroexpressed rHWTX-I was eluted at an acetonitrile concentration of 30.4% (upper). A native HWTX-I sample was also loaded into the same C18 column as a control, although the gradients used were slightly different; native HWTX-I was eluted at an acetonitrile concentration of 30.6% (lower). Eluted rHWTX-I was collected, vacuum dried, and subjected to further analysis.

Figure 4B shows the mass spectrometry analysis results of the RP-HPLC purified product. The molecular mass was determined to be 3750.69 Da, compared with the theoretical molecular weight of 3756.45 of native HWTX-I (calculated by adding all 33 amino acid residues' mass weights together); a 6-dalton difference represents the formation of three disulfide bonds by six cysteines. Based on the mass spectrometry analysis, the overall yield of purified rHWTX-I was about 0.7 mg/L.

### Purified rHWTX-I possesses native electrophyiological properties of HWTX-I

**Figure 5:**
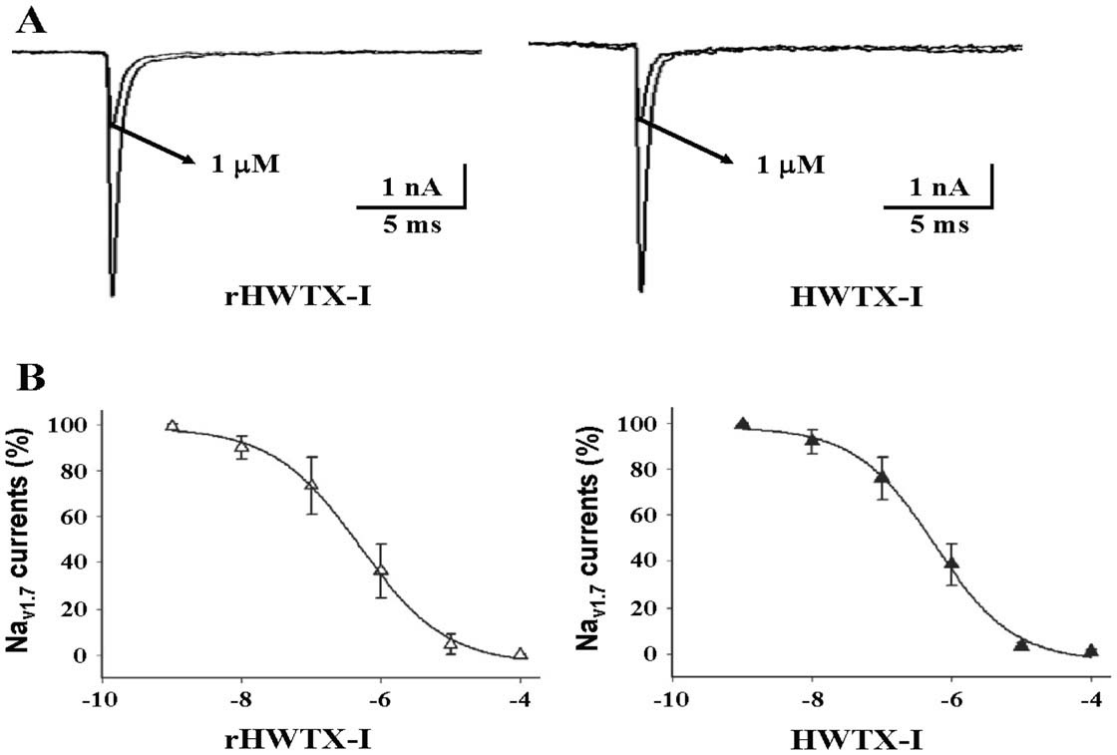
Effects of rHWTX-I and HWTX-I on wild-type hNav_1.7_ channels expressed in HEK293 cells. A) Effects of rHWTX-I (left) or native HWTX-I (right) on currents through hNav_1.7_ channels that were elicited by depolarization to −10 mV from a holding potential of −100 mV. The toxin concentration was 1 µmole/L. B) Effects of rHWTX-I (left) or native HWTX-I (right) on dose-response inhibitory curves of hNav_1.7_ channels. Data points shown as mean ± S.E. (each from three to five experimental cells). The IC_50_ values of hNav_1.7_ in response to rHWTX-I and native HWTX-I were 640 nmole/L and 630 nmole/L, respectively.

The electrophysiological properties of purified rHWTX-I were analyzed using a whole-cell patch-clamp assay. As shown in Figure 5A, 1 µmole/L of expressed rHWTX-I demonstrated identical inhibitory activity to that of native HWTX-I at the same concentration; both of them were able to reduce the hNa_v1.7_ current to 33–36%. The calculated IC_50_ of rHWTX-I was 640 nmole/L, a value almost identical to that of native HWTX-I, which was 630 nmole/L (Figure 5B).

## Discussion

It has been estimated that there are more than 1 million currently existing spider species. Based on a conservative estimate, the potential number of unique spider venom peptides could be more than 12 million [19]. Spider venoms, as well as venoms from snakes, frogs, scorpions, sea anemones, and cone snails, have been widely studied in past decades and have led to the discovery of a large number of bioactive peptides. The biological diversity of animal venom peptides refined by the evolutionary process makes them preoptimized molecules that could be readily used in either structure/function studies of ion channels/receptors, or the development of modern drugs.

It was proposed recently that venomics, a high-throughput approach based on a combination of MS and molecular biology methods, can be used as a new paradigm for venom exploration [20]. However, for each and every unique bioactive peptide identified by venomics or other cutting-edge technologies, a complete structural/functional characterization is necessary before it can be used either as a research tool or a drug model molecule. In these cases, the production of a sufficient quantity of the peptide remains the greatest bottleneck.

There are three major strategies of venom peptide production: classical biochemical preparation, direct peptide synthesis, and the expression of peptide-coding nucleotides. Since most venomous animals are of very small size, the utilization of bioassay-guided fractionation technologies is greatly confined due to the limited amount of venom available. It is also notable that most bioactive peptides identified from invertebrates comprise about 15 to 70 amino acid residues and are reticulated by several disulfide bridges [21]. Therefore, the direct synthesis of these peptides is challenged by not only low efficiency and high cost, but also the oxidative folding of disulfide-rich peptides.

Various systems were used previously for the recombinant expression of small peptide toxins with disulfide bridges, including bacterial systems (mostly *E. coli*) [8], [22], [23], [24], yeast [25], and cultured insect cells [26]. Although it is hard to predict which system is suitable for the expression of a specific bioactive peptide, the *E. coli* system, due to its ease of handling and reasonable product/cost ratio, is always the first choice for most researchers.

As mentioned above, recombinant HWTX-I was expressed in the cytoplasm of *E. coli* cells in fusion with GST [8]. Although the original yield of the fusion protein was more than 10 mg/L culture, the rHWTX-I released by enzyme digestion exhibited very low bioactivity. Mass spectrometry analysis demonstrated that no disulfide bridge formed, yet the formation of the three disulfide bonds in the natural form is critical to HWTX-I's functions. It was reported that the cytoplasm of *E. coli* cells contains high levels of reduced glutathione (approximately 5 mmole/L) [27]; therefore, the potential of the cytoplasm is too reducing for most disulfide bonds to form. Compared with the cytoplasm, the periplasm of *E. coli* cells provides a relatively non-reducing environment that allows disulfide bridges to form [28]. It was reported that several peptide toxins, including Dendrotoxin K and Huwentoxin-XI, were functionally expressed in the periplasm of *E. coli* with the formation of disulfide bridges found in their natural forms [29], [30].

Compared with other reported procedures for the expression of disulfide bridge-rich peptides in the periplasm of *E. coli*, two major improvements were significant in this work. First, DsbC was used in this system to lead the periplasmic expression of rHWTX-I. As a disulfide interchange protein, DsbC was reported to favor the formation of disulfide bridges in their natural forms [31], [32]. Second, instead of the commonly used IPTG induction protocol, an auto-induction medium was used in these procedures. Compared with IPTG induction, auto-induction with lactose does not affect the normal growth of *E. coli* cells and induces the expression of a target protein more gently. In addition, since the transportation of DsbC fusion protein to the periplasm is mainly controlled by the SecYEG translocase, the auto-induction procedure effectively precludes the formation of inclusion bodies that might block the Sec-dependent pathway [33], [34], [35], [36].

In summary, the procedures reported here provide an efficient method for the expression of HWTX-I with native bioactivities. It is expected that this method will be widely used for the expression of more bioactive peptides with multiple disulfide bridges.
